# A Large Open Pangenome and a Small Core Genome for Giant Pandoraviruses

**DOI:** 10.3389/fmicb.2018.01486

**Published:** 2018-07-10

**Authors:** Sarah Aherfi, Julien Andreani, Emeline Baptiste, Amina Oumessoum, Fábio P. Dornas, Ana Claudia dos S. P. Andrade, Eric Chabriere, Jonatas Abrahao, Anthony Levasseur, Didier Raoult, Bernard La Scola, Philippe Colson

**Affiliations:** ^1^Microbes Evolution Phylogenie et Infections (MEϕI), Institut Hospitalo-Universitaire Méditerranée Infection, Assistance Publique – Hôpitaux de Marseille, Institut de Recherche pour le Développement, Aix-Marseille Université, Marseille, France; ^2^Departamento de Microbiologia, Instituto de Ciências Biológicas, Universidade Federal de Minas Gerais, Belo Horizonte, Brazil

**Keywords:** pandoravirus, giant virus, megavirales, pangenome, core genome

## Abstract

Giant viruses of amoebae are distinct from classical viruses by the giant size of their virions and genomes. Pandoraviruses are the record holders in size of genomes and number of predicted genes. Three strains, *P. salinus*, *P. dulcis*, and *P. inopinatum*, have been described to date. We isolated three new ones, namely *P. massiliensis*, *P. braziliensis*, and *P. pampulha*, from environmental samples collected in Brazil. We describe here their genomes, the transcriptome and proteome of *P. massiliensis*, and the pangenome of the group encompassing the six pandoravirus isolates. Genome sequencing was performed with an Illumina MiSeq instrument. Genome annotation was performed using GeneMarkS and Prodigal softwares and comparative genomic analyses. The core genome and pangenome were determined using notably ProteinOrtho and CD-HIT programs. Transcriptomics was performed for *P. massiliensis* with the Illumina MiSeq instrument; proteomics was also performed for this virus using 1D/2D gel electrophoresis and mass spectrometry on a Synapt G2Si Q-TOF traveling wave mobility spectrometer. The genomes of the three new pandoraviruses are comprised between 1.6 and 1.8 Mbp. The genomes of *P. massiliensis*, *P. pampulha*, and *P. braziliensis* were predicted to harbor 1,414, 2,368, and 2,696 genes, respectively. These genes comprise up to 67% of ORFans. Phylogenomic analyses showed that *P. massiliensis* and *P. braziliensis* were more closely related to each other than to the other pandoraviruses. The core genome of pandoraviruses comprises 352 clusters of genes, and the ratio core genome/pangenome is less than 0.05. The extinction curve shows clearly that the pangenome is still open. A quarter of the gene content of *P. massiliensis* was detected by transcriptomics. In addition, a product for a total of 162 open reading frames were found by proteomic analysis of *P. massiliensis* virions, including notably the products of 28 ORFans, 99 hypothetical proteins, and 90 core genes. Further analyses should allow to gain a better knowledge and understanding of the evolution and origin of these giant pandoraviruses, and of their relationships with viruses and cellular microorganisms.

## Introduction

Giant viruses of amoebae are distinct from classical viruses by many features, primarily by the giant size of their virions and genomes (Colson et al., 2017a). The first to be discovered was Mimivirus, in 2003 (La Scola et al., 2003). Since then, giant viruses that were described were classified into two viral families and several new putative viral groups (Colson et al., 2017b). Their remarkable characteristics and expanding diversity have raised many questions about their origin and evolution. Notably, these giant viruses display several traits that are hallmarks of cellular organisms, including the encoding of several translation components by their genomes. Pandoraviruses were discovered in 2013 (Philippe et al., 2013). The first pandoravirus was isolated from a marine sediment layer of a river on a coast of Chile (Philippe et al., 2013), the second one from a freshwater pond in Australia (Philippe et al., 2013), and the third one from contact lenses and their storage case fluid of a keratitis patient in Germany (Scheid et al., 2014). These viruses hence appear to be cosmopolitan, and pandoravirus-like sequences were detected in metagenomes generated from water and soil samples collected worldwide (Verneau et al., 2016; Kerepesi and Grolmusz, 2017; Brinkman et al., 2018) as well as from mosquitoes (Temmam et al., 2015; Atoni et al., 2018), biting midges (Temmam et al., 2015), and simian bushmeat and human plasma (Verneau et al., 2016; Temmam et al., 2017). Pandoraviruses became, and still are, the record holders in size of viral genomes and number of predicted genes. In addition, their virions exhibit a weird morphology for viruses, being ovoid, surrounded by a tegument-resembling structure, and devoid of recognizable capsid (Philippe et al., 2013). As for the mimiviruses, they had been for years mingled with intra-amoebal eukaryotic parasites (Scheid et al., 2014).

The isolation of all giant viruses of amoebae until now was made possible through the use of amoebae of the genus *Acanthamoeba* or *Vermamoeba* as culture support (Khalil et al., 2017). This culture strategy has been considerably optimized during the past 15 years, with, notably, the implementation of high-throughput amoebal co-culture protocols (Khalil et al., 2017). Such approach was recently used to discover new giant viruses of amoebae in Brazil (Dornas et al., 2015). Consequently, three new pandoraviruses were isolated in 2015–2016 from water collected from a Soda lake and from soil samples (Dornas et al., 2015). We describe here the genomes of these three new giant viruses and the pangenome of pandoraviruses based on these three new isolates and the three previously described strains, namely *Pandoravirus dulcis*, *Pandoravirus salinus* (Philippe et al., 2013), and *Pandoravirus inopinatum* (Scheid, 2016).

## Materials and Methods

### Virus Isolation, Production, and Purification

After collection, samples were stored at −80°C and then co-cultured on *Acanthamoeba castellanii*, as previously described (Andreani et al., 2016). The three samples induced amoebal lysis, and then were subcultured to produce the new virus isolates. Viruses were then purified and concentrated by centrifugation (Andreani et al., 2016).

### Genome Sequencing

The viral genomes were sequenced on the Illumina MiSeq instrument (Illumina, Inc., San Diego, CA, United States) by using both paired-end and mate-pair strategies for *P. massiliensis* and *P. braziliensis*, and paired-end strategy only for *P. pampulha*. Genomic DNA was quantified by a Qubit assay with the high-sensitivity kit (Life technologies, Carlsbad, CA, United States). DNA paired-end libraries were constructed with 1 ng of each genome as input with the Nextera XT DNA sample prep kit (Illumina, Inc., San Diego, CA, United States), according to the manufacturer’s recommendations. Automated cluster generation and paired-end sequencing with dual index reads were performed in a single 39-h run in 2 × 250 bp. Paired-end reads were trimmed and filtered according to read qualities. The mate-pair library was prepared with 1.5 μg of genomic DNA. Genomic DNA was simultaneously fragmented and tagged with a mate-pair junction adapter. The library profile and the concentration were visualized on a high-sensitivity bioanalyzer labchip (Agilent Technologies Inc., Santa Clara, CA, United States). In each construction, libraries were normalized at 2 nM and pooled, denaturated, and diluted to reach a concentration of 15 pM, before being loaded onto the reagent cartridge, then onto the instrument along with the flow cell. Automated cluster generation and sequencing run were performed in a single 39-h run generating 2 × 151-bp long reads. The quality of the genomic data was analyzed by FastQC^1^.

### Genome Assembly

The three pandoravirus genomes were assembled using CLC genomics v.7.5^2^ with default parameters. The assembly of the *P. massiliensis* genome provided nine scaffolds. Gaps were filled and scaffolds were then reordered using both Sanger sequencing and three different assembly tools used in combination, including A5, Velvet, and ABySS (Simpson et al., 2009; Zerbino, 2010; Tritt et al., 2012). The genome of *P. braziliensis* was assembled into seven scaffolds, which were then reordered into two scaffolds by using similarity searches and synteny bloc detection with the closest available genomes. Long-range PCR was performed to resolve the linear or circular organization of the two scaffolds. The genome of *P. pampulha* was assembled into 45 scaffolds that were reordered and fused to form one fragment, using the same strategy than for *P. braziliensis*.

### Transcriptome Sequencing of *Pandoravirus massiliensis*

The transcriptome of *P. massiliensis* was analyzed at the following times: 30 min (t0), then 2 (t2h), 4 (t4h), 6 (t6h), and 8 h (t8h) after inoculation of the virus on *A. castellanii* in Peptone Yeast Glucose browth medium. At each time point, the co-culture was centrifuged then immediately frozen at -80°C. RNA was extracted with the RNeasy mini kit (Qiagen, Hilden, Germany). After cDNA generation by RT-PCR, libraries were constructed with the Nextera XT DNA sample prep kit. cDNA was quantified by a Qubit assay with the high-sensitivity kit. To prepare the paired-end library, dilution was performed to require 1 ng of each genome as input. The “tagmentation” step fragmented and tagged the DNA. Then, limited cycle PCR amplification (12 cycles) completed tag adapters and introduced dual-index barcodes. The library profile was validated on an Agilent 2100 Bioanalyzer with a DNA high-sensitivity labchip (Agilent Technologies Inc., Santa Clara, CA, United States), and the fragment size was estimated to be 1.5 kbp. After purification on AMPure XP beads (Beckman Coulter Inc., Fullerton, CA, United States), libraries were normalized on specific beads according to the Nextera XT protocol (Illumina, Inc.). Normalized libraries were pooled for sequencing on the MiSeq instrument. Automated cluster generation and paired-end sequencing with dual index reads were performed in a single 39-h run in 2 × 250 bp. Total information of 3.6 Gb was obtained from a 370 k/mm^2^ cluster density with a cluster passing quality control filters of 95.7% (6,901,000 passed filtered clusters). Within this run, the index representation for *P. massiliensis* infection kinetic was respectively determined to be 2.5, 9.4, 3.7, 15.1, and 0.6%. Finally, paired-end reads were trimmed and filtered according to the read qualities.

### Proteome Analysis of *Pandoravirus massiliensis*

#### Preparation of the Total Proteins of the Virus

Samples were rapidly lysed in DTT solubilization buffer (2% SDS, 40 mM Tris–HCl, pH 8.0, 60 mM DTT) with brief sonication. The 2D Clean-Up kit eliminated nucleic acids, salts, lipids, and other reagents not compatible with immunoelectrophoresis.

#### Two-Dimensional Gels

Analysis of the 1D gel electrophoresis was performed with the Ettan IPGphor II control software (GE Healthcare). For the 2D gel electrophoresis, buffer (50 mM Tris–HCl, pH 8.8, 6 M urea), 30% glycerol, 65 mM dithiothreitol reducing solution, alkylating solution of iodoacetamide at 100 mM, and SDS-PAGE gel at 12% acrylamide were used. The polyacrylamide gel was prepared in the presence of TEMED, a polymerization agent, and ammonium persulfate. Sodium dodecyl sulfate at 2% was used to denature proteins. Migration was carried out under the action of a constant electric field of 25 mA for 15 min followed by 30 mA for ≈5 h. Silver nitrate was used for protein staining. Proteins of interest were recovered by cutting the gel.

#### Mass Spectrometry

For global proteomic analysis, the protein-containing solution was subjected to dialysis and trypsin digestion. Dialysis was carried out using Slide-ALyzer 2K MWCO dialysis cassettes (Pierce Biotechnology, Rockford, IL, United States) against a solution of 1 M urea and 50 mM ammonium bicarbonate pH 7.4, twice, during 4 h, and one night. Protein digestion was carried out by adding 2 μg of trypsin solution (Promega, Charbonnières, France) to the alkylated proteins, with incubation at 37°C overnight in a water bath. The digested sample was then desalted using detergent columns (Thermo Fisher Scientific, Illkirch, France) and analyzed by mass spectrometry on a Synapt G2Si Q-TOF traveling wave mobility spectrometer (Waters, Guyancourt, France) as described previously (Reteno et al., 2015). An internal protein sequence database was used that was built primarily with two types of amino acid sequences: (i) sequences obtained by translating *P. massiliensis* open reading frames (ORFs); (ii) sequences obtained by translating the whole genome into the six reading frames then fragmenting the six translation products into 250 amino acid-long sequences with a sliding step of 30 amino acids. Contiguous sequences positive for peptide detection were fused and re-analyzed.

### Genome Annotation

Gene predictions were performed using GeneMarkS and Prodigal softwares, and results were merged (Besemer and Borodovsky, 2005; Hyatt et al., 2010). ORFs shorter than 50 amino acids were discarded. Predicted proteins were annotated by comparative genomics by using BLASTp searches against the NCBI GenBank non-redundant protein sequence database (nr), with an e-value threshold of 1e–3. ORFans were defined as ORFs without homolog in the nr database considering as thresholds an e-value of 1e–3 and a coverage of the query sequences by alignments of 30%. Functional annotation was refined by using DeltaBLAST searches (Boratyn et al., 2012). Best reciprocal hits were detected by the Proteinortho program with an amino acid identity percentage and a coverage thresholds of 30 and 70%, respectively (Lechner et al., 2011). The core genome and the pangenome were estimated by clustering predicted proteins with CD-HIT (Huang et al., 2010) using 30 and 50% as thresholds for sequence identity and coverage, respectively. Transfer RNAs (tRNAs) were predicted using Aragorn (Laslett and Canback, 2004).

### Transcriptomic Analysis for *Pandoravirus massiliensis*

Reads generated from the RNA extracts were mapped on the assembled genome by using the bowtie2 software with default parameters (Langmead et al., 2009; Langmead, 2010; Langmead and Salzberg, 2012). Mapping results were analyzed using the HTseq-count software, with the union mode (Anders et al., 2015). Only “aligned” results were taken into account. Predicted ORFs were considered as transcribed if at least 10 reads were aligned.

### Search for Transposable Elements

Miniature inverted repeat transposable elements (MITE) previously identified in the *P. salinus* genome were searched for by using the BLASTn program with an evalue threshold of 1e-3 (Sun et al., 2015). MITE are DNA transposons whose size ranges between 100 and 600 bp and that require transposition enzymes from other, autonomous transposable elements.

### Phylogenetic Analyses and Hierarchical Clustering

Phylogeny reconstruction was performed based on the DNA-dependent RNA polymerase subunit 1. Amino acid sequences were aligned using Muscle (Edgar, 2004). The phylogenetic tree was built using FastTree with default parameters (Price et al., 2010). Hierarchical clustering was performed with the Mev program (Chu et al., 2008) based on the presence/absence patterns of pandoravirus genes that are homologous to clusters of orthologous groups of proteins previously delineated for nucleocytoplasmic large DNA viruses and giant viruses of amoebae (NCVOGs) (Yutin et al., 2013).

## Results

**FIGURE 1:**
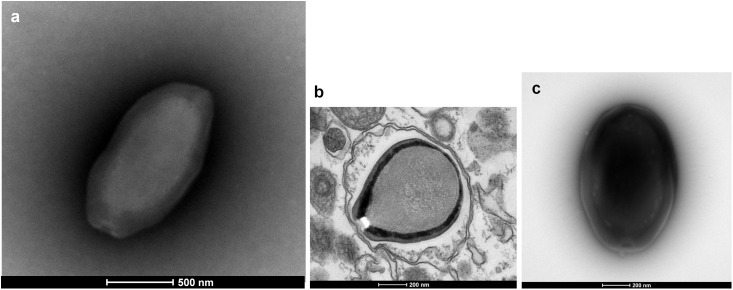
Electron microscopy pictures of pandoravirus isolates by negative staining **(a,c)** or after inclusion **(b)**. **(a)** *Pandoravirus massiliensis*; **(b)** *Pandoravirus pampulha*; **(c)** *Pandoravirus braziliensis*.

Three new pandoravirus isolates were obtained from soil and water samples collected in Brazil in 2015–2016. Two pandoraviruses were isolated in 2015 from soil samples collected from Pampulha lagoon and Belo Horizonte city. A third pandoravirus was isolated in 2016 from a Soda lake (Soda lake2). These new viruses were named *Pandoravirus massiliensis* strain BZ81 c (**Figure 1a**), *Pandoravirus pampulha* strain 8.5 (**Figure 1b**), and *Pandoravirus braziliensis* strain SL2 (**Figure 1c**), respectively.

For the *P. massiliensis* genome, 403,592 reads were obtained by the mate-pair sequencing, with a length ranging from 35 to 251 nucleotides, and the average quality per read was 28 and 37 for the forward and the reverse sequences, respectively. For the paired-end sequencing, 269,656 reads were obtained with a length ranging from 35 to 251 nucleotides; the average quality score per read was 37 for the forward and the reverse sequences, respectively. The *P. massiliensis* genome (EMBL Accession no. OFAI01000000) was assembled in two scaffolds of 1,593,057 and 2,489 bp, and was predicted to encode 1,414 proteins (**Table 1**). Mean size (±SD) of these proteins is 299 ± 228 amino acids. Median size is 218 amino acids. A total of 25% of these predicted proteins are smaller than 136 amino acids, and 25% are larger than 397 amino acids, among which 15 proteins are larger than 1,000 amino acids. Among these 1,414 proteins, 786 (56%) have a homolog in the NCBI GenBank nr database (using a BLASTp e-value threshold of 1e-3), and 628 (44%) are ORFans (ORFs with no significant homolog in the NCBI nr database). Among ORFs that have a homolog in nr, 744 (95%) have genes from previously described pandoraviruses as best BLASTp hits. Two genes encode for Pro-tRNA and Cys-tRNA. A total of 74 ORFs have a significant BLASTp hit with a NCVOG. A total of 310 ORFs were found to be paralogous genes. Finally, 425 ORFs (30% of the gene content) belong to the strict core genome delineated for the six pandoraviruses. For the *P. pampulha* genome, a total of 864,982 reads were obtained, with a length ranging from 35 to 251 nucleotides; the average quality score per read was 37 for the forward and the reverse strands, respectively. The *P. pampulha* genome (EMBL Accession no. OFAJ01000000) was assembled in a single scaffold of 1,676,092 bp, and predicted to encode 2,368 proteins and two tRNA, a Pro-tRNA, and a Trp-tRNA (**Table 1**). Mean size of these proteins is 237 ± 219 amino acids. Among these ORFs, 58% have no homolog in the nr database. Among the 989 ORFs that have a homolog in nr, 974 (98%) have genes from previously described pandoraviruses as best BLASTp hits. A total of 72 ORFs have a hit with a NCVOG. We detected that 407 ORFs (17%) are paralogs. Finally, 417 ORFs (18%) were found to belong to the strict core genome of the pandoraviruses. For the *P. braziliensis* genome, a total of 542,496 reads with a length ranging from 35 to 251 nucleotides were obtained for the paired-end run; the average quality score per read was 37 for the forward and the reverse sequences, respectively. For the mate-pair run, a total of 2,194,091 reads were obtained, with a length ranging from 35 to 251 nucleotides; the average quality score per read was 37 for the forward and the reverse strands, respectively. The assembly of the *P. braziliensis* genome (EMBL Accession no. OFAK01000000) provided two scaffolds with a length of 1,828,953 and 21,873 bp (**Table 1**). A total of 2,693 proteins were predicted, their mean size being 215 ± 212 amino acids. Three genes encode a Leu-tRNA, a Pro-tRNA, and a Pyl-tRNA. ORFans represent 67% of the ORF set. Among the 892 ORFs that have a homolog in nr, 872 (98%) have genes from previously described pandoraviruses as best BLASTp hits. Moreover, 72 ORFs are homologous to a NCVOG. We detected that 437 ORFs are paralogs. Finally, 428 ORFs (16%) were found to be shared with the five other pandoraviruses. All these three genomes were found to be linear double-stranded DNA, as described previously for *P. salinus*, *P. dulcis*, and *P. inopinatum*. Thus, here, PCR amplification performed with the attempt to test the circularity of the genome failed. BLASTp hits were found in nr for hundreds of additional short ORFs predicted in the genomes of *P. pampulha* and *P. braziliensis*, but e-values were >1e-3, and only short fragments from these sequences were usually involved in alignments obtained with these hits.

**Table 1:** Main features of the six pandoravirus genomes.

Virus	Genome length	Number of scaffolds	Number of predicted proteins	GC%	Proportion of the gene content that belongs to the core genome (%) (number of proteins)
*Pandoravirus dulcis*	1,908,520	1	1,487	63.7	29.4 (437)
*Pandoravirus salinus*	2,473,870	1	2,541	61.7	17.3 (440)
*Pandoravirus inopinatum*	2,243,110	1	1,839	60.7	26.9 (495)
*Pandoravirus pampulha*	1,676,092	1	2,368	63.9	17.6 (416)
*Pandoravirus braziliensis*	1,850,826	2	2,693	59.0	15.4 (415)
*Pandoravirus massiliensis*	1,595,546	2	1,414	60.1	29.3 (414)

**FIGURE 2:**
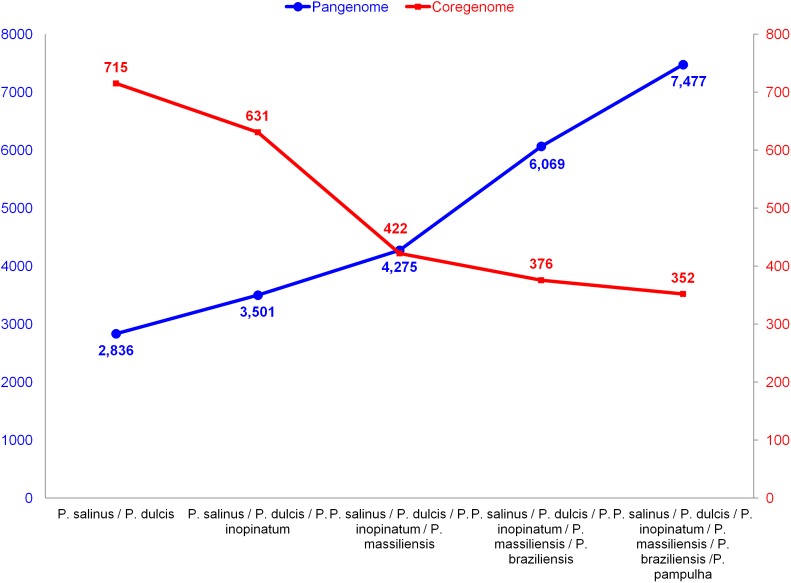
Evolution of the size of the pangenome and core genome of pandoraviruses with the description of each of the six pandoravirus isolates.

**FIGURE 3:**
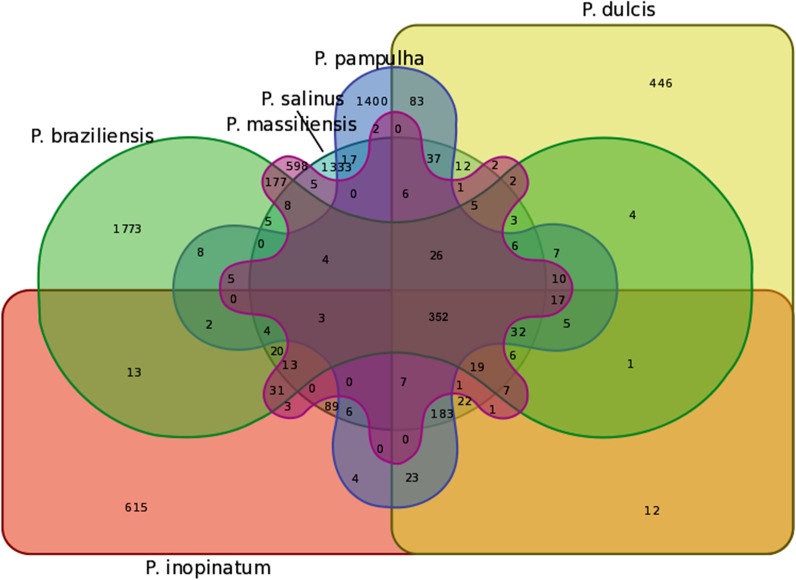
Venn diagram of genes shared and not shared between the gene contents of the six pandoravirus isolates. Venn diagram was built using the following online tool: http://bioinformatics.psb.ugent.be/webtools/Venn/.

**FIGURE 4:**
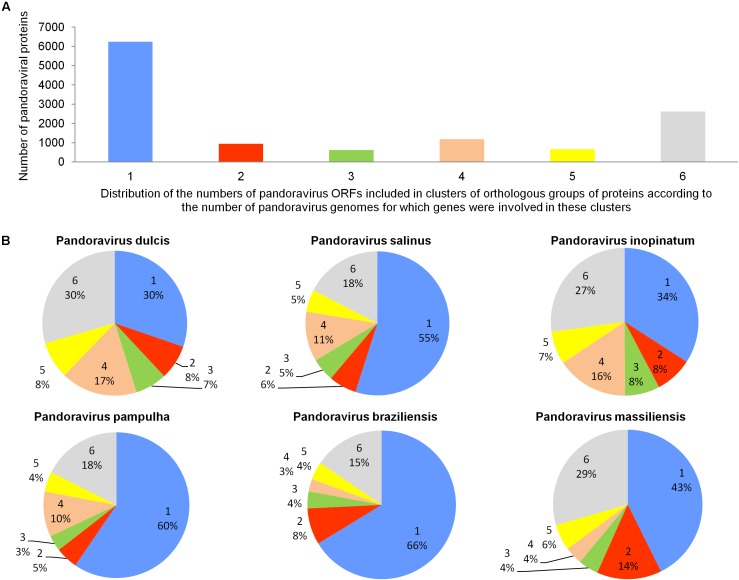
Distribution of the number of pandoravirus ORFs included in clusters of orthologous groups of proteins according to the number of pandoravirus genomes for which genes were involved in these clusters **(A)** and proportion for each pandoravirus genome of the genes involved in clusters including genes from one to six pandoraviruses **(B)**.

The pangenome size delineated for these three new pandoravirus genomes and the three previously described pandoravirus genomes reaches 7,477 gene comprising clusters or unique genes (**Figures 2**, **3**). Among them, 6,108 (82%) encompass a single predicted gene (**Figure 4**). A total of 427 clusters (5.7%) are composed of two representative sequences and 163 clusters (2.2%) are composed of three representative sequences. The “strict” core genome represents 4.7% of the pangenome. It includes 352 clusters comprising 2,617 pandoravirus proteins, each of these clusters encompassing at least one predicted protein from each of the six pandoravirus isolates. The ratio core genome/pangenome is thus less than 0.05 and the proportion for each individual virus of the gene content that belongs to the core genome is comprised between 15.4 and 29.4%. When considering the proteins involved in best reciprocal hits with an identity >30% and a query sequence coverage >70%, a total of 208 clusters of proteins (1.6% of the full cluster set) encompassed at least one protein of each of the six pandoravirus isolates. Besides, a homolog was found in the gene content of all six pandoraviruses for a NCVOG in 403 cases.

Only 13% of the *P. massiliensis* transcripts were detected during the first 4 h post-infection of the amoeba by this virus. In contrast, more than two-thirds of the transcripts (69%) were detected 6 h post-infection of the amoeba, and 18% of them were detected 8 h post-infection. A total of 359 *P. massiliensis* ORFs (25% of the gene content) were detected by transcriptomics taking into account all reads at any time post-infection, with a mean coverage of 50 reads/ORF along the whole genome. Among these 359 ORFs, three (ORFs 1, 1,350, and 1,364) had a particularly high coverage, greater than 1,200 reads/ORF (1,592, 1,243, and 1,234, respectively). When removing these three ORFs, the mean coverage of transcripts along the genome decreased to 39 reads/ORF. Two of these three ORFs are hypothetical proteins and were detected in the five other pandoraviruses. Nevertheless, the product of only one of these two ORFs was found by proteomics. Strikingly, this ORF is harbored by the 2,489 bp-long genomic fragment. The second of these two ORFs is contiguous to two other highly transcribed genes (with 425 and 552 mapped reads). The third most transcribed ORF is a collagen triple helix encoding protein, also found by proteomics. Finally, a total of 210 of the 359 transcribed ORFs (58%) is part of the core genome; while 60% of the ORFs that are part of the core genome were transcribed. Conversely, only 149 (14%) of the 1,062 *P. massiliensis* ORFs that do not belong to the core genome were transcribed.

A total of 162 ORFs were found by proteomic analysis of the *P. massiliensis* virions. Among them, 90 proteins (55%) are part of the core genome. Conversely, a protein was found in *P. massiliensis* virions for only 72 (7%) of the 1,062 ORFs that did not belong to the core genome. In addition, the products of 28 ORFans and 99 hypothetical proteins were part from these 162 proteins detected by proteomic analyses. The most abundant peptides found in the *P. massiliensis* virions match with 37 proteins, which include 12 ORFan gene products; 19 hypothetical proteins; a trimeric LpxA-like enzyme motif-containing protein; a translation initiation inhibitor belonging to the YJGF family; a thioredoxin-like fold motif-containing protein; a laminin G domain-containing protein; a collagen triple helix repeat domain-containing protein; and an ankyrin repeat-containing protein. A concordance between transcriptomic and proteomic data was found for 89 ORFs (**Supplementary Table S1**). These ORFs include 2 ORFans and 61 hypothetical proteins, all found in other pandoraviruses. The other ORFs with functional annotations have a pandoravirus protein as their most similar sequence. These ORFs notably encode an acid phosphatase class b; a C1q domain-containing protein; two casein kinases; a cathepsin c1-like peptidase; a trypsin-like serine protease; a disulfide isomerase motif-containing protein; a DNA pol III gamma/tau subunit-like domain containing protein; an FAD/FMN-containing dehydrogenase; a hexapeptide repeat-containing protein; a histidine phosphatase motif-containing protein; a laminin G domain-containing protein; a lipase/esterase; an NAD-dependent amine oxidase; an oxidoreductase; an SMC ATPase domain-containing protein, SMC proteins being ATPases involved in chromosome organization and dynamics; a thioredoxin-like fold motif-containing protein; two translation initiation inhibitors belonging to the YJGF family; and a trimeric LpxA-like enzyme motif-containing protein (bacterial transferase). Of note, for the five genes predicted to encode DNA-dependent RNA polymerase subunits, transcripts were only detected for those encoding subunits 1 and 2 and no protein was detected by proteomics. Finally, among *P. massiliensis* ORFans, 26 (4.1%) were found to be transcribed, and a similar number (28; 4.5%) were found to encode proteins detected in virions.

**FIGURE 5:**
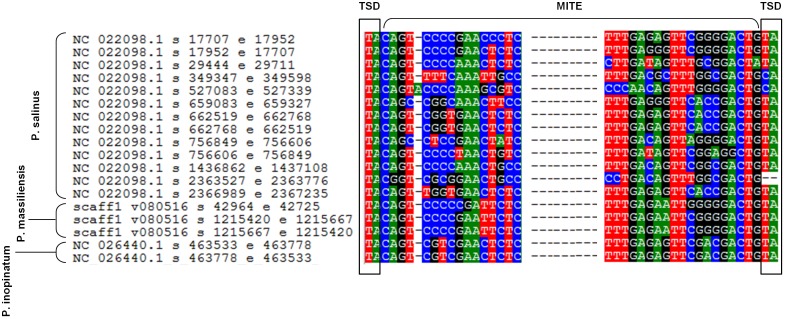
Alignment by muscle of the 13 full-length miniature inverted repeat transposable elements (MITEs) described in the genome of *Pandoravirus salinus* (Sun et al., 2015) and those detected in the genomes of *P. massiliensis* and *P. inopinatum*. Alignment is limited to regions including the flanking target site duplication (TSD) sequences and the start and end of the MITEs.

Sequences similar to MITEs were identified through BLAST searches, in all six genomes of pandoraviruses, albeit their number varied considerably according to the genome. Thus, eight different matches with MITEs were identified in the *P. massiliensis* genome, which displayed a nucleotide identity varying between 78 and 100% with a MITE identified in *P. salinus* (Sun et al., 2015). Seven matches with MITEs were detected in the *P. inopinatum* genome, which were 76–100% identical with a *P. salinus* MITE. Five matches with MITEs were detected in *P. braziliensis* and *P. dulcis*, which were 75–98 and 76–95% identical with a MITE identified in *P. salinus*, respectively. Finally, four matches with MITEs were identified in the *P. pampulha* genome, which were 82–98% identical with a *P. salinus* MITE. However, when considering full-length MITE copies described for the *P. salinus* genome, 3 and 2 such full-length MITEs were detected in the genomes of *P. massiliensis* and *P. inopinatum*, respectively (**Figure 5**). These sequences did not cluster together according to the isolate (**Supplementary Figure S1**).

**FIGURE 6:**
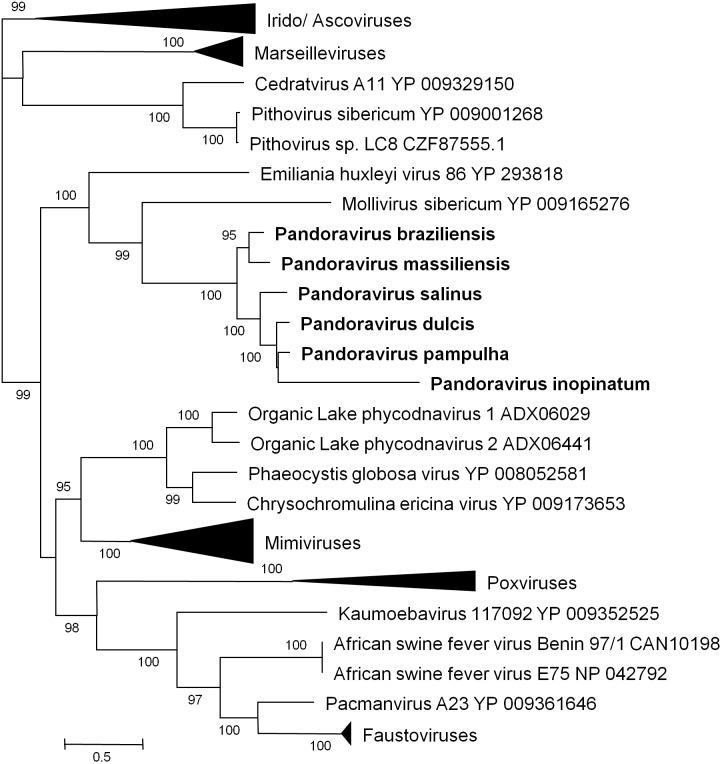
Phylogenetic reconstruction based on amino acid sequences of the DNA-dependent RNA polymerase subunit 1 from representatives of megavirales. Phylogenetic tree was drawn using the maximum likelihood model with the FastTree program (Price et al., 2010).

**FIGURE 7:**
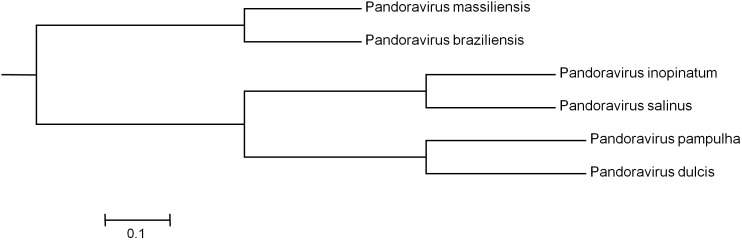
Hierarchical clustering based on the presence/absence patterns of clusters of orthologous groups of proteins of megavirales members in the genomes of pandoraviruses.

**FIGURE 8:**
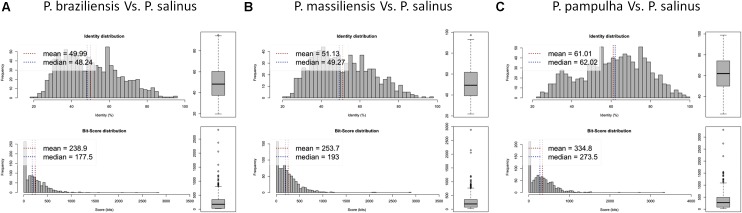
Average amino acid identity between ORFs predicted for pandoravirus genomes. For each comparison, estimates were obtained using both best hits and reciprocal best hits between two sets of proteins from a pandoravirus newly described here and *Pandoravirus salinus*. **(A)** *Pandoravirus braziliensis* versus *Pandoravirus salinus*, **(B)** *Pandoravirus massiliensis* versus *Pandoravirus salinus*, **(C)** *Pandoravirus pampulha* versus *Pandoravirus salinus*.

Phylogenetic reconstruction based on the RNA polymerase subunit 1 showed that *P. massiliensis* and *P. braziliensis* were closely related (**Figure 6**). Hierarchical clustering showed congruent results with a close relationship between *P. massiliensis* and *P. braziliensis* (**Figure 7**). In addition, mean amino acid identities between orthologous proteins of *P. salinus* and *P. massiliensis* or *P. braziliensis* were similar (mean values, 50.0 and 51.1%, respectively), and lower than the mean amino acid identity between orthologous proteins of *P. salinus* and *P. pampulha* (61.0%) (**Figure 8**). Taken together, on the basis of phylogenetic analysis, the presence/absence patterns of clusters of orthologous groups of proteins of Megavirales members, and amino acid identity of orthologous proteins, two major groups can be delineated for these six pandoravirus isolates. The first group is comprised by *P. massiliensis* and braziliensis, and the second group is comprised by *P. salinus*, *P. dulcis*, *P. pampulha*, and *P. inopinatum*.

**FIGURE 9:**
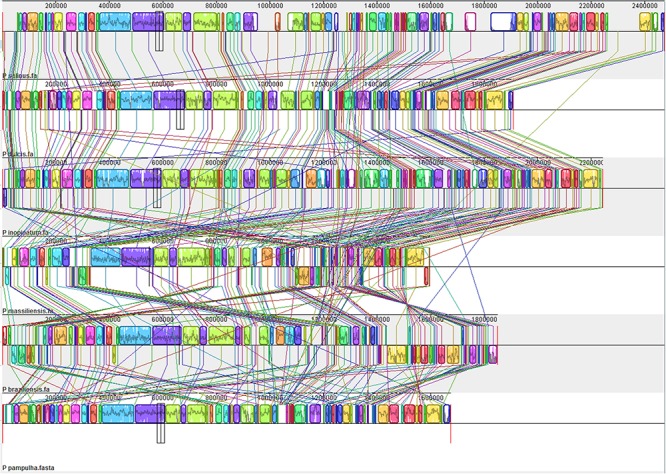
Whole genome alignment of pandoravirus genomes by the MAUVE program (Darling et al., 2010).

**FIGURE 10:**
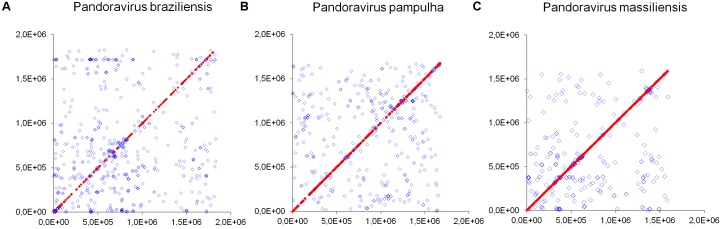
Distribution of core genes and paralogous genes along the pandoravirus genomes. **(A)** *Pandoravirus braziliensis*; **(B)** *Pandoravirus pampulha*; **(C)** *Pandoravirus massiliensis*. Core genes are indicated by red dots; paralogous genes are indicated by blue diamonds.

Comparison of genome architecture and co-linearity showed a general tendency among the different pandoravirus genomes for a greater co-linearity around the first third of the genome alignement by the MAUVE software, displaying large blocks with a high level of nucleotide identity (**Figure 9**). Besides, dot plots constructed separately for the three new pandoravirus isolates described here on the basis of their gene content showed a considerable number of paralogous genes, and the scattering of core genes along the whole genome length (**Figure 10**). Paralogous genes mostly consisted in three groups of proteins with ankyrin repeat motifs, F-box domains, and MORN-repeats. Finally, the gene of *P. salinus* recently described as a putative candidate for encoding a capsid protein (ps_862) (Sinclair et al., 2017) was detected in the genomes of *P. braziliensis*, *P. massiliensis*, and *P. pampulha*. However, the product of this gene was not found in the proteome of *P. massiliensis* virions.

## Discussion

We delineated here the pangenome and core genome of pandoraviruses based on six viruses, including three new isolates from Brazil. Our findings indicate that pandoraviruses, first described in 2013, are likely common in water and soil samples worldwide, as is the case for mimiviruses and marseilleviruses. The various pandoravirus isolates described to date were isolated from three continents in Chile, Australia, Germany, and Brazil (Philippe et al., 2013; Scheid et al., 2014; Dornas et al., 2016; Andrade et al., 2018). Moreover, our results indicate that pandoraviruses currently form a homogenous viral group, regarding both their morphology and their genome organization and content.

Our findings further point out that these giant viruses are currently those with the largest genomes, which range in size from 1.59 Mbp (for *P. massiliensis*) to 2.47 Mbp (for *P. salinus*). Far smaller genomes have been described for other giant viruses, namely pithoviruses (Legendre et al., 2014; Levasseur et al., 2016) and cedratviruses (Andreani et al., 2017; Bertelli et al., 2017). Indeed, genome size is 0.61–0.68 Mbp for pithoviruses and 0.57–0.59 Mbp for cedratviruses. This is intriguing as the size of pithovirus and cedratvirus virions, which have a similar morphology than pandoravirus virions and a similar tegument-resembling structure delineating the particle, is similar to those of pandoravirus virions, or even larger for pithoviruses (up to 1.5–2.5 μm compared to c.a. 1 μm for pandoraviruses) (Legendre et al., 2015; Okamoto et al., 2017). Such discrepancies between genome and virion sizes have been rarely described (Cui et al., 2014; Brandes and Linial, 2016).

We noted here a great size of the pandoravirus pangenome (comprised by 7,477 unique genes or clusters of genes), compared with that delineated most recently for mimiviruses (2,869 clusters) (Assis et al., 2017) and marseilleviruses (665 clusters) (Dornas et al., 2016). Furthermore, expansion of this pangenome since 2013, while taking into account the three new pandoravirus genomes described here, suggests it is still open with a mean increase of 28% at each new genome annotation. Conversely, a major finding of our pangenome analysis is that pandoraviruses have a core genome size that is limited relatively to the number of genes predicted in each of their genomes. Thus, the proportion for each individual virus of the gene content that belongs to the core genome is lower than 30% and as low as 15%. Compared to the 352 clusters of genes described for the pandoravirus core genome, mimiviruses core genome comprises 267 clusters of genes based on 21 described genomes with a size ranging between 1,017 and 1,259 Mbp (Assis et al., 2017) and the marseillevirus core genome comprises 202 clusters of genes based on 8 described genomes with a size ranging between 0.347 and 0.386 Mbp (Dornas et al., 2016).

Strikingly, a significant number of pandoravirus predicted ORFs have no homolog in the international databases and no predicted functions. This proportion of ORFans remains greater than for other giant viruses of amoebae (Colson et al., 2017b). The *P. massiliensis* transcriptomic and proteomic analyses showed that at least a small proportion of these ORFan genes are transcribed and encode for proteins. This highlights that most of the gene armentarium involved in the structure, metabolism, and replication of these pandoraviruses is currently unknown, as is the case for all other giant viruses of amoebae. We also noted that coding capacity differed greatly from one pandoravirus genome to another. Thus, *P. braziliensis* harbors the biggest gene content with a total of 2,693 predicted genes and a coding capacity of 1.45 gene/kbp. In contrast, *P. dulcis*, with a genome of similar size, is predicted to encode only 1,502 genes, corresponding to a coding capacity of 0.79 gene/kbp. Regarding the genomes of the three new pandoraviruses, the mean size of their genes varies greatly, from 215 to 299 amino acids. Moreover, the gene contents of the three new pandoraviruses differ in terms of proportions of ORFans, ranging betweeen 44 and 67%.

The presence of MITEs in the pandoravirus genomes are another evidence of the presence of transposable elements in the genomes of giant viruses of amoebae. Previously, transpovirons were described in mimivirus genomes, and genomes of virophages were found to integrate as provirophages in the genomes of these mimiviruses (Desnues et al., 2012). Moreover, introns were described in genomes of several giant viruses of amoebae (Desnues et al., 2012; Philippe et al., 2013; Colson et al., 2017b). Taken together, all these elements correspond to a mobilome for these giant viruses (Desnues et al., 2012). In addition to full-length MITEs, we detected several sequences in the different pandoravirus genomes that match with full-length MITEs. They might correspond to degraded MITE sequences or to different elements. Besides, two ribonuclease H-like domain motif-containing proteins were detected as part of the transcriptome of *P. massiliensis*. This deserves being mentioned since the presence of ribonuclease H in the genomes of giant virus has been recently studied and suggested to be associated with sequence integration (Moelling et al., 2017).

In summary, our knowledge of the pandoravirus diversity continues to expand (Andrade et al., 2018). Further analyses should allow to gain a better knowledge and understanding of the evolution and origin of these giant pandoraviruses, and of their relationships with viruses and cellular microorganisms.

## Author Contributions

PC, BS, DR, and SA designed the experiments. SA, PC, JAN, EB, AO, FD, AA, and AL contributed to the data and performed the experiments. SA, PC, JAB, EC, AL, DR, and BS analyzed the data. PC, BS, and SA wrote the manuscript.

## Conflict of Interest Statement

The authors declare that the research was conducted in the absence of any commercial or financial relationships that could be construed as a potential conflict of interest.

## Footnotes

1 https://www.bioinformatics.babraham.ac.uk/projects/fastqc/

2 https://www.qiagenbioinformatics.com/

## Supplementary Material

FIGURE S1 Molecular phylogenetic analysis of miniature inverted repeat transposable elements (MITEs) detected in the genomes of *P. salinus*, *P. massiliensis* and *P. inopinatum*. The tree was built with nucleotide sequences from **Figure 5**, using the Maximum Likelihood method. Blue squares indicate sequences of *P. salinus*; green circles indicate sequences of *P. massiliensis*; gray losanges indicate sequences of *P. inopinatum*.

TABLE S1 *Pandoravirus massiliensis* predicted genes for which a transcript has been detected by transcriptomics and a product has been detected by proteomic.
