# Supplementary figures and images for: A Large Open Pangenome and a Small Core Genome for Giant Pandoraviruses
Source: Front Microbiol. 2018 Jul 10;9:1486. doi: 10.3389/fmicb.2018.01486 (PMC6048876; doi:10.3389/fmicb.2018.01486)

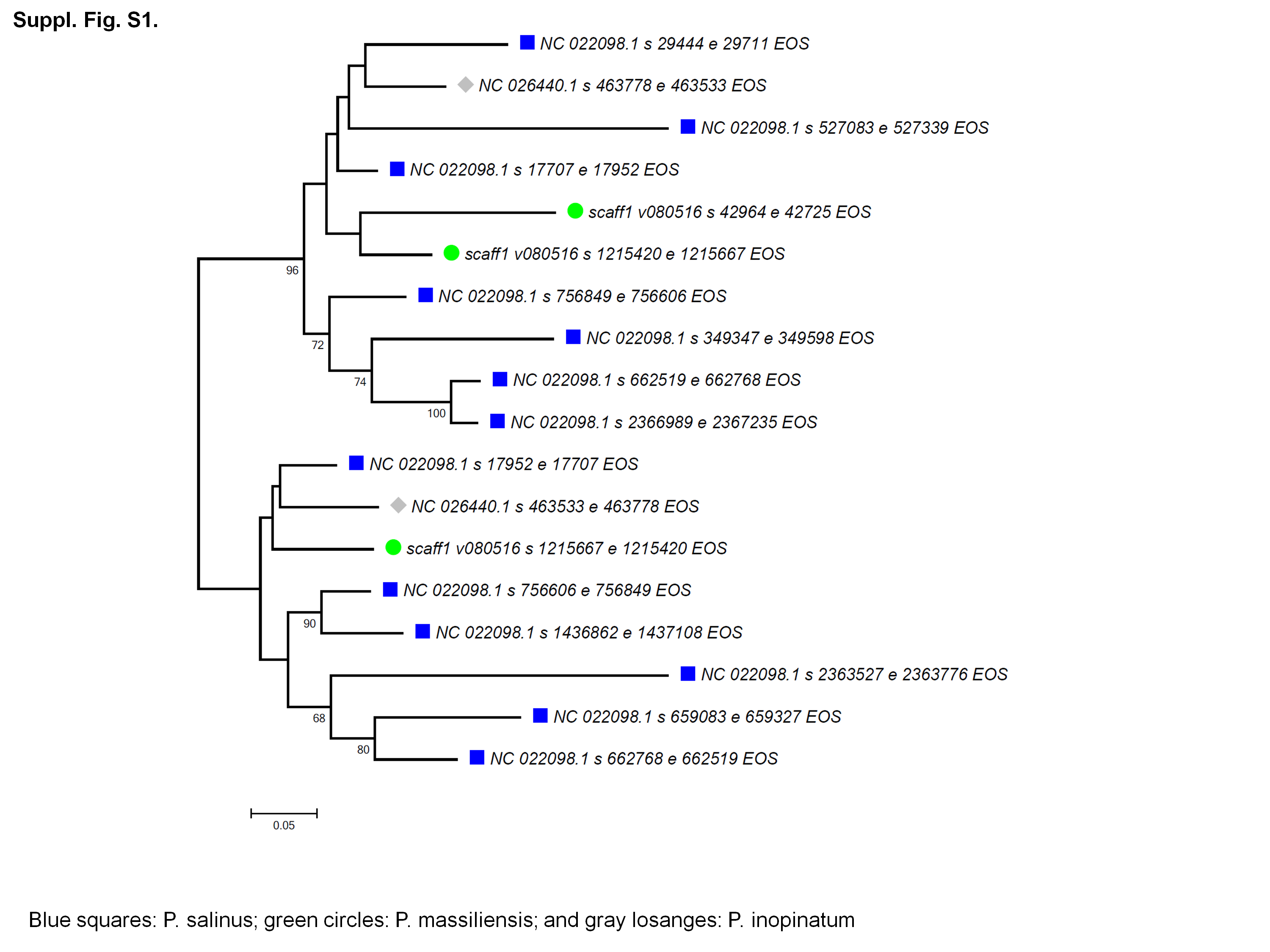

Supplement: FIGURE S1 — Molecular phylogenetic analysis of miniature inverted repeat transposable elements (MITEs) detected in the genomes of P. salinus, P. massiliensis and P. inopinatum. The tree was built with nucleotide sequences from Figure 5, using the Maximum Likelihood method. Blue squares indicate sequences of P. salinus; green circles indicate sequences of P. massiliensis; gray losanges indicate sequences of P. inopinatum. [file Image_1.TIF]
